# Role and Mechanism of Sialic Acid in Alleviating Acute Lung Injury through In Vivo and In Vitro Models

**DOI:** 10.3390/foods13182984

**Published:** 2024-09-20

**Authors:** Dan Li, Fangyan Li, Yaping Zhou, Yiping Tang, Zuomin Hu, Qi Wu, Tiantian Xie, Qinlu Lin, Hanqing Wang, Feijun Luo

**Affiliations:** 1 Hunan Key Laboratory of Grain-Oil Deep Process and Quality Control, Hunan Key Laboratory of Forestry Edible Resources Safety and Processing, National Research Center of Rice Deep Processing and Byproducts, College of Food Science and Engineering, Central South University of Forestry and Technology, Changsha 410004, China; ld1764409498@163.com (D.L.); zyp4265@163.com (Y.Z.); tangyiping@126.com (Y.T.); huzuomin97100214@163.com (Z.H.); xiaowuu1998@163.com (Q.W.); xtt04160530@163.com (T.X.); t20081475@csuft.edu.cn (Q.L.); 2 Hunan Engineering Research Center of Full Life-Cycle Energy-Efficient Buildings and Environmental Health, School of Civil Engineering, Central South University of Forestry and Technology, Changsha 410004, China; 17736602213@163.com

**Keywords:** sialic acid, acute lung injury, transcriptome, anti-inflammatory activity, anti-oxidant effect

## Abstract

Excessive inflammatory reactions are the most important pathological injury factor in acute lung injury (ALI). Our recent study found that sialic acid had an anti-colitis effect. In this study, the effect of sialic acid (SA) on acute lung inflammation was investigated. A lipopolysaccharide (LPS)-induced ALI animal model and LPS-stimulated HUVEC cell model were used to evaluate the anti-inflammatory effect of SA and study its molecular mechanisms. Compared with the LPS group, the lung index of the SA group decreased from 0.79 ± 0.05% to 0.58 ± 0.06% (LPS + 50 SA) and 0.62 ± 0.02% (LPS + 100 SA), with *p* < 0.01, suggesting that SA could improve the pulmonary edema of mice and alleviate LPS-induced lung injury. Transcriptome research identified 26 upregulated genes and 25 downregulated genes involved in the protection of SA against ALI. These genes are mainly related to the MAPK and NF-κB signaling pathways. Our study also proved that SA markedly downregulated the expression of inflammatory factors and blocked the JNK/p38/PPAR-γ/NF-κB pathway. Meanwhile, SA treatment also upregulated the expression of HO-1 and NQO1 in ALI mice. In vitro, SA obviously repressed the expressions of inflammatory cytokines and the JNK/p38-NF-κB/AP-1 pathway. SA also regulated the expression of oxidative stress-related genes through the Nrf2 pathway. Taken together, SA exhibits a protective role by modulating the anti-inflammatory and anti-oxidation pathways in ALI, and it may be a promising candidate for functional foods to prevent ALI.

## 1. Introduction

Respiratory disease, as a prevalent lung disorder, is one of the key concerns in human health. Acute lung injury (ALI) is a common form of respiratory system disease and may subsequently cause the occurrence of acute respiratory dysfunction syndrome (ARDS) in severe conditions [1]. It has become a global health problem with high mortality and morbidity rates [2]. The pathogenesis of ALI is immensely complex, and increasing attention has been gained for finding a cure for it [3,4,5]. Pharmacological treatment is the major therapy approach, though it is limited by drug resistance and adverse effects. Naturally occurring ingredients have shown potential functional activities to improve this health situation [6]. Therefore, research regarding the natural bioactive component for adjunct prevention is of great significance.

The edible bird’s nest (EBN) is a popular type of food known for its nutritional value in China. It is believed to possess outstanding health-promoting functions, such as an antiviral property [7], bone strengthening [8], an anti-aging effect [9], neuroprotective activity [10], and immunity regulation [11]. These health benefits are attributed to the bioactive composition of the EBN. EBNs are rich in carbohydrates, proteins, peptides, amino acids, ash, and fat [9]. Accumulating studies have demonstrated that sialic acid (SA) is the central compound in EBN glycoprotein, and it is responsible for human health [12,13]. Interest in the biological functions and mechanisms of SA has grown continually, and it could be a promising component for functional and medicinal products.

**Figure 1:**
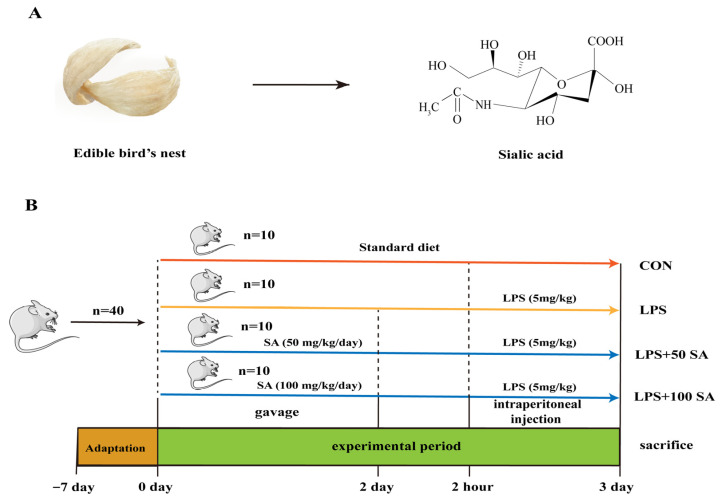
**Schematic illustration of the structure of SA obtained from EBNs and the experimental protocol.** SA specially refers to Neu5Ac in EBNs (**A**). The ICR mice were assigned to four groups randomly (*n* = 10 per group)—the CON group, LPS group, LPS + 50 SA group, and LPS + 100 SA group—after 1 week of acclimation. All groups were reared with standard diets. In the LPS + 50 SA group and LPS + 100 SA group, mice were pre-gavaged with SA (50 mg/kg/day and 100 mg/kg/day) for two consecutive days. Two hours after the last gavage, the mice were injected with 5 mg/kg LPS (LPS group, LPS + 50 SA group, and LPS + 100 SA group) or normal saline (CON group) intraperitoneally. At 24 h after treatment with LPS, the mice were sacrificed for further analysis (**B**). CON: control; EBN: edible bird’s nest; LPS: lipopolysaccharide; Neu5Ac: N-acetylneuraminic acid; SA: sialic acid.

SA, an acidic sugar bound to the glycan terminal of EBN glycoprotein, is also known as N-acetylneuraminic acid (Neu5Ac) in EBNs. It is a negatively charged nine-carbon monosaccharide derived from neuraminic acid (Figure 1A). With the in-depth investigation of SA, various physiological functions have been explored. For instance, SA could promote brain development and enhance intellectual capacity [14]. Its strong negative charge is conducive to the absorption of nutrients with positive charges (e.g., Ca^2+^ and Mg^2+^) [15]. SA is also associated with tumorigenesis, immune evasion, and metastasis [16]. What is more, the protective roles of SA, including anti-virus action [7], regulation of microbial communities [17], and anti-inflammatory and anti-oxidant activities [18,19], were illustrated. Interestingly, SA could maintain the homeostasis of organisms and contribute to pulmonary developmental biology [20]. These studies confirmed the potential functional activities of SA. However, there is no report which provides direct evidence of the protective role and molecular mechanism of SA against ALI.

In this work, the effect of SA obtained from EBNs on ALI was assessed with an lipopolysaccharide (LPS)-induced ALI animal model and an LPS-stimulated human umbilical vein endothelial cell (HUVEC) model. The possible molecular mechanism was further investigated with transcriptome analysis, bioinformatics techniques, and molecular biology methods. The findings elucidate the protective effect of SA against ALI and facilitate the development of functional foods for ALI.

## 2. Materials and Methods

### 2.1. Chemicals and Reagents

SA (purity: 99%, white powder) was bought from Chengdu Push Biotechnology Co., Ltd. (Chengdu, China). SA was dissolved in distilled water, and various concentrations of SA solutions were homogenized with a turbine mixer for 2 min. LPS was ordered from Sigma Aldrich Co., Ltd. (Shanghai, China). RPMI-1640 medium was obtained from Gibco BRL (Carlsbad, CA, USA). Fetal bovine serum (FBS) was provided from Inner Mongolia Opcel Biotechnology Co., Ltd. (Hohhot, China) A radioimmunoprecipitation assay (RIPA) buffer solution was purchased from Servicebio Co., Ltd. (Wuhan, China). Nuclear and cytoplasmic protein extraction kit was obtained from Beyotime Biotechnology (Shanghai, China). A PrimeScript™ RT kit was brought from the Takara Company (Takara, Shiga, Japan). Polyclonal antibody tumor necrosis factor-α (TNF-α, CAT. #6945S), interleukin-6 (IL-6, CAT. #12912S), interleukin-1β (IL-1β, CAT. #12242S), nuclear factor kappa-light-chain-enhancer of activated B cells (NF-κB) p65 (CAT. #12629S), activator protein 1 (AP-1, CAT. #9165S), and mitogen-activated protein kinase (MAPK) family antibody (JNK, CAT. #9252S; p-JNK, CAT. #4668S; p38, CAT. #9212S; p-p38, CAT. #4511S) were provided by Cell Signaling Technology (Danver, MA, USA). Nuclear factor erythroid 2 related factor 2 (Nrf2, CAT. #66504-1-lg), Kelch-like ECH-associated protein 1 (Keap1, CAT. #60027-1-lg), NADH quinone oxidoreductase 1 (NQO1, CAT. #67240-1-lg), and heme oxygenase-1 (HO-1, CAT. #67643-1-lg) antibody were obtained from Proteintech Company (Chicago, IL, USA). β-Actin, anti-mouse IgG Horseradish Peroxidase (HRP) conjugate, and anti-rabbit IgG HRP conjugate were obtained from Promega Corporation (Madison, WI, USA). Other chemical reagents used in this investigation were of analytical grade.

### 2.2. Animal Experiments

A total of 40 ICR male mice (8 weeks old; obtained from Hunan SJA Laboratory Animal Co., Ltd., Changsha, China) were reared with a laboratory basal diet and water under the condition of 12 h dark/light cycles, temperatures of 25  ±  2 °C, and humidity of 60 ± 5%. All animal experimental procedures were approved by the Hunan Laboratory Animal Center (Hunan Drug Safety Evaluation Research Center Co., Ltd., Changsha, China) (IACUC-2021[5]068, Liuyang, Changsha, China) and followed the Guidelines for the Care and Use of Experimental Animals. After 1 week of acclimation, these mice were assigned to four groups randomly (n = 10): the control (CON) group, LPS-induced ALI (LPS) group, LPS + low dose (50 mg/kg/day) SA (LPS + 50 SA) group, and LPS + high dose (100 mg/kg/day) SA (LPS + 100 SA) group. Mice in the LPS + SA groups were supplemented with SA-water solutions (50 and 100 mg/kg/day) intragastrically for two consecutive days. Two hours after the last gavage, the mice in the LPS and LPS + SA groups were injected with 5 mg/kg LPS intraperitoneally. The control mice were given normal saline intragastrically at the same dosage. At 24 h after treatment with LPS, the mice were anesthetized and sacrificed (Figure 1B), and the samples were collected for further analysis.

### 2.3. Biochemical Assessments

In each group, the lung sections were weighed and homogenized with normal saline. The samples were centrifuged at 3500 rpm for 20 min, and the supernatants were collected for biochemical analysis. The superoxide dismutase (SOD), malondialdehyde (MDA), and glutathione peroxidase (GSH-Px) contents were determined by following the assay kits (Nanjing Jiancheng Bioengineering Institute, Nanjing, China).

### 2.4. Histopathology Analysis

For histological examination, the lung tissues were resected and fixed in paraformaldehyde solution for 24 h. After dehydration with ethanol, the specimens were made transparent with xylene and inlaid into paraffin. Then, these samples were sectioned into pieces and dyed using hematoxylin and eosin (H&E). Histopathological images of the lung tissues were acquired with a microscope (Nikon Corporation, Tokyo, Japan).

### 2.5. Transcriptomics Analysis

RNA isolation and RNA sequencing analysis of the lung tissues (about 100 mg/sample) were conducted by Majorbio Bio-Pharm Technology Co., Ltd. (Shanghai, China). The total lung tissue RNA was extracted with Transzol-Up reagent according to the manufacturer’s instructions. Genomic DNA was removed by using DNase I (TaKara). Then, RNA quality was evaluated using 2100 Bioanalyser (Agilent Technologies Co., Ltd., Santa Clara, CA, USA) and quantified with an ND-2000 (Thermo Fisher Scientific, Waltham, MA, USA). High-quality RNA samples (OD260/280 = 1.8~2.2, OD260/230 ≥ 2.0, RIN ≥ 8.0, 28S:18S ≥ 1.0, >1 μg) were used to construct a sequencing library. The RNA sequencing library was generated using a TruSeqTM RNA sample preparation kit from Illumina (San Diego, CA, USA). Then, RNA sequencing was carried out on the Illumina NovaSeq 6000 platform. The raw data were quality controlled by fastp to obtain clean reads [21,22]. HISAT2 (v2.2.1) software was used to separately align clean reads to reference genomes [23]. The gene expression and gene abundances were calculated using transcripts per million readings and RSEM [24]. In this study, genes with a *p* value <0.05 and fold change >1.5 or <0.667 were considered significantly differentially expressed genes (DEGs). The functional enrichment analysis of DEGs was performed using the Gene Ontology (GO) and Kyoto Encyclopedia of Genes and Genomes (KEGG) databases. Protein interactions were built and visualized using the STRING (v12.0) website and Cytoscape (3.10) software.

### 2.6. Cell Culture

For cellular experiments, HUVEC cells were provided by the Institute of Cell Resource Center of the Chinese Academy of Science (Shanghai, China). The HUVEC cells were maintained in RPMI Medium 1640 containing 10% FBS at 37 °C in a 5% CO_2_ atmosphere. After cell attachment, the HUVEC cells were incubated with different concentrations of SA and then induced with LPS.

### 2.7. Cell Viability Assay

The cell viability was determined through an MTS assay. The HUVEC cells were seeded in 96 well plates and maintained for 24 h at 37 °C in a 5% CO_2_ incubator. Then, different concentrations of SA (0, 6.25, 12.5, 25, 50, and 100 μg/mL) were added to the cells. After incubation for 12 h, the cell morphology was imaged with an inversion fluorescence microscope (Nikon 80i, Tokyo, Japan). Then, the supernatant was discarded, and the MTS solution was added to the cell followed by incubating for about 30 min at 37 °C in the dark. The cytotoxicity of the SA was measured based on the absorbance at 490 nm using a microplate reader (Thermo Multiskan SPECTRUM, Thermo Fisher Scientific, Waltham, MA, USA). The experiments were repeated 3 times, and the cell viability was presented as a percentage.

### 2.8. Real Time-Quantitative PCR

Lung tissues (about 100 mg/sample) were ground with a liquid nitrogen precooled mortar. The total RNA was extracted using Transzol-Up reagent (Transgen, Beijing, China) according to the manufacturer’s protocol. The quality and concentration of the total RNA samples were tested with a Nano-Drop Ultramicro spectrophotometer instrument (Thermo Fisher Scientific, Shanghai, China). The high-quality RNA sample was used to synthesize the first-strand cDNA with High-Capacity cDNA Reverse Transcription Kits (Applied Biosystems, Foster City, CA, USA). A real-time quantitative polymerase chain reaction (RT-qPCR) assay was performed utilizing the CFX96 Real Time PCR system (Applied Biosystems, USA) according to the manufacturer’s instructions for the SYBR ® Select Master Mix (Applied Biosystems). The relative expressions of the target genes were assessed based on the 2^−△△Ct^ (RQ) method. The amplification conditions and analysis of gene expressions were described previously [25]. The primer sequences are shown in Table 1.

### 2.9. Total Protein Extraction and Western Blot Analysis

The lung tissues (about 100 mg/sample) were ground into powder in a liquid nitrogen precooled mortar. Then, the samples were suspended in RIPA buffer solution, phenylmethylsulfonyl fluoride, protease inhibitor cocktail, and phosphatase inhibitors. The protein was obtained from the supernatant solution after centrifugation of the suspension at 4 °C and 13,000 rpm/min for 15 min. In addition, HUVEC cells were treated with different concentrations of SA (0, 25, 50, and 100 μg/mL) for 2 h, after which LPS was added to induce inflammation. Total protein was extracted according to the above methods. The cell nucleus and cytoplasm proteins were obtained according to the guidelines of nuclear/cytoplasmic protein extraction kit. The protein concentrations were determined via a bicinchoninic acid (BCA) protein assay kit (Beyotime Biotechnology Co., Ltd., Shanghai, China) following the protocol. The prepared protein samples were dissolved in sodium dodecyl sulfate (SDS) loading buffer and heated for 15 min under 95 °C. Then, the mixed protein samples were subjected to SDS-polyacrylamide gel electrophoresis for separation and subsequently transferred to the polyvinylidene difluoride membrane. The membranes were blocked with 5% bovine serum albumin for about 1 h at room temperature. Next, the membranes were incubated with the primary antibody overnight at 4 °C. These membranes were washed 3 times (10 min per time) at 25 °C, followed by incubation with the secondary antibody of anti-mouse or anti-rabbit IgG HRP conjugate at 25 °C for 1–2 h. After being washed 3 times again, the immunoreactive proteins were tested via the ECL Plus™ western blot (WB) detection system (Pierce, Rockford, IL, USA), and the signals were observed in the gel imaging system (Chemi Doc XRS+, Bio-Rad Laboratories, Inc., Hercules, CA, USA). The relative quantity of the target protein compared with the control group was determined by calculating the integrated optical density of each band.

### 2.10. Statistical Analysis

All experiments were conducted in triplicate, and statistical analyses were carried out with SPSS 22.0 software (SPSS, Chicago, IL, USA). Data were expressed as the mean ± standard deviation (SD). Before performing the *t*-test, analysis of variance (ANOVA) was used to assess the data distribution and variance homogeneity of the data from different groups. A *p* value <0.05 was regarded as statistically significant.

## 3. Results

### 3.1. SA Improved the Macroscopic Phenotypes of the LPS-Stimulated Mice

**Figure 2:**
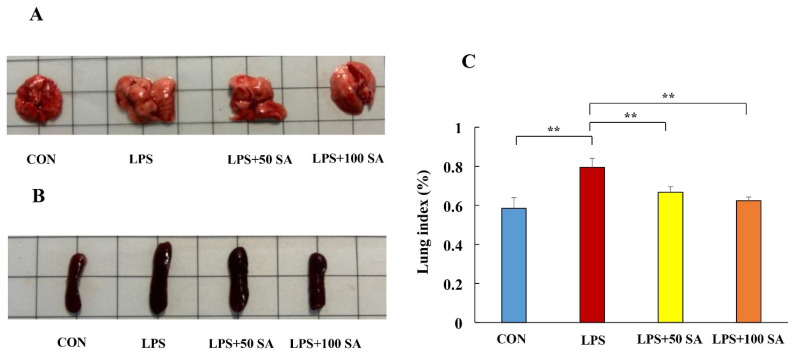
**Effect of SA supplementation on the macroscopic phenotypes of LPS-induced mice.** Mice were treated with LPS or SA, and the lung and spleen tissues were collected for analysis at the end of the experiment. (**A**) Representative images of lung tissue. (**B**) Representative images of spleen. (**C**) Lung index (lung weight divided by body weight). CON: control; LPS: lipopolysaccharide; LPS + 50 SA: LPS + 50 mg/kg/day SA; LPS + 100 SA: LPS + 100 mg/kg/day SA; SA: sialic acid. The values are presented as mean ± SD. CON group vs. LPS group or LPS group vs. LPS + 50 SA group and LPS + 100 SA group. ** *p* < 0.01.

Changes in the lung and spleen tissues are critical indicators of ALI. After the experiments, the lung and spleen tissues were photographed and measured to investigate the protective role of SA in LPS-stimulated mice. The mice in the LPS group had a marked swollen lung and what appeared to be severe pulmonary lesions, suggesting the ALI model’s success (Figure 2A). Nevertheless, SA intragastric administration significantly improved LPS-induced lung injury. It could partially restore the lung index (lung weight divided by body weight). The lung indexes in the CON group, LPS group, LPS + 50 SA group, and LPS + 100 SA group were 0.58 ± 0.06%, 0.79 ± 0.05%, 0.67 ± 0.03%, and 0.62 ± 0.02% (*p* < 0.01), respectively (Figure 2C). The spleen plays the immunomodulatory role in the body, and it was also impaired in the LPS-treated mice. The size of the spleen was greater in the LPS group, but splenomegaly was ameliorated by SA treatment (Figure 2B). These results clearly reveal that SA had a beneficial effect on LPS-induced ALI.

### 3.2. SA Increased the Antioxidant Ability in ALI

**Figure 3:**
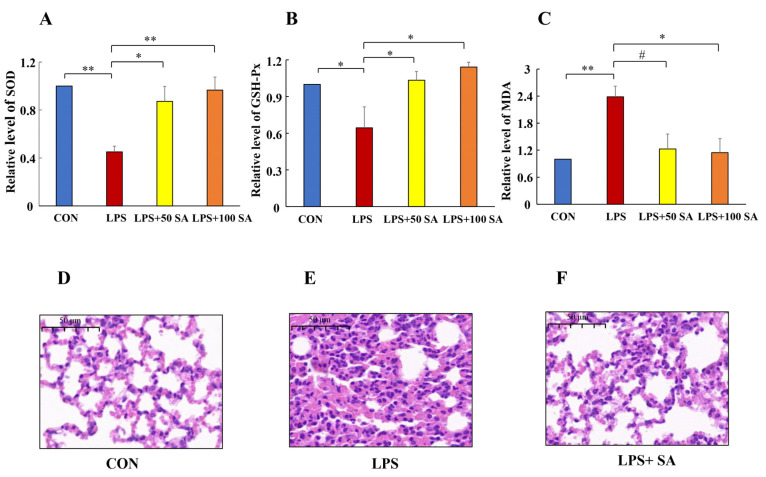
**Effect of SA supplementation on the biochemical indexes and histopathological changes of lung tissue.** The MDA content and the activities of SOD and GSH-Px were detected by assay kits. (**A**) Effect of SA on the pulmonary SOD activity. (**B**) Effect of SA on the GSH-Px activity of lung tissues. (**C**) Effect of SA on the pulmonary MDA level. The pathological features of the lung tissue are illustrated by H&E staining. The CON group revealed no remarkable lesions (**D**). Pulmonary tissues in LPS-treated group displayed significantly inflammatory cell infiltration and congestion (**E**). Administering 100 mg/kg SA mitigated lung pathological symptoms (**F**). CON: control; GSH-Px: glutathione peroxidase; H&E: hematoxylin and eosin; LPS: lipopolysaccharide; LPS + 50 SA: LPS+ 50 mg/kg/day SA; LPS + 100 SA/LPS + SA: LPS + 100 mg/kg/day SA; MDA: malondialdehyde; SA: sialic acid; SOD: superoxide dismutase. Results are shown as mean ± SD. CON group vs. LPS group or LPS group vs. LPS + 50 SA group and LPS + 100 SA group. * *p* < 0.05. ** *p* < 0.01. # *p* > 0.05.

Oxidative stress could reflect the status of a lung injury. The MDA content and the activities of SOD and GSH-Px were evaluated in this study. The oxidative damage can be characterized by these indexes. As shown in Figure 3A,B, a considerable decrease in SOD and GSH-Px activities was detected in the LPS group. The SOD activity decreased the most after LPS treatment, reducing by 54.85% (*p* < 0.01), while the SOD activity in the SA protected group was much higher than that in the LPS group. In addition, the GSH-Px activity was reduced by 35.56% (*p* < 0.05) in the LPS-treated mice. SA administration observably reversed the decline in GSH-Px activity. In comparison with the CON group, the content of MDA significantly increased (1.38 fold) in the model group. SA treatment showed suppression of the MDA level. In particular, the level of MDA was successfully decreased by 52.10% after 100 mg/kg SA supplementation (Figure 3C). These figures could illustrate that SA intervention apparently relieved the oxidative stress in the LPS-induced mice.

### 3.3. Effect of the SA on the Histopathologic Changes in LPS-Induced ALI

To investigate the effect of SA on the LPS-induced lung damage, the pathological features of lung were illustrated by H&E staining. The histological lung sections of the CON group revealed no remarkable lesions, and the LPS group showed severe lung pathological symptoms. A normal pulmonary structure and thin alveolar wall were apparently present in the CON group (Figure 3D). LPS stimulation increased inflammatory cell infiltration, fibrosis, congestion, and the width of alveolar septum significantly. Aside from that, a reduction in the alveolar number occurred in the LPS group (Figure 3E). In contrast, a 100 mg/kg SA gavage could reduce inflammatory infiltration, congestion, and fibrosis (Figure 3F). Taken together, SA exerted a protective effect on LPS-induced lung injury.

### 3.4. SA Regulated the Gene Expression Profiles of the Lung Tissues

**Figure 4:**
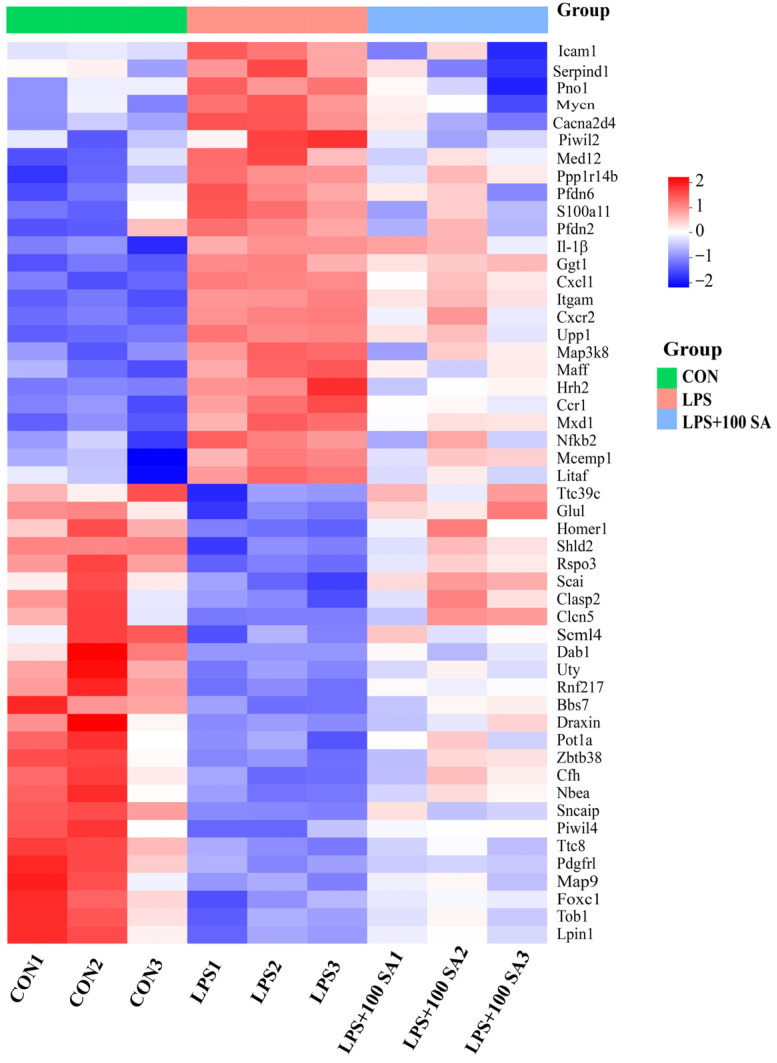
**Transcriptome analysis of pulmonary tissues from mice.** DEGs were screened among the CON group, LPS group, and LPS + 100 SA group. The genes upregulated in LPS/CON and downregulated in LPS + 100 SA/LPS groups and vice versa were considered the DEGs of SA against ALI, with a *p* value < 0.05 and fold change  >1.5 or <0.667 regarded as the threshold for significant DEGs. In all, 51 DEGs were screened and analyzed by hierarchical clustering. ALI: acute lung injury; CON: control; DEGs: differentially expressed genes; LPS: lipopolysaccharide; LPS + 100 SA: LPS + 100 mg/kg/day SA; SA: sialic acid.

**Figure 5:**
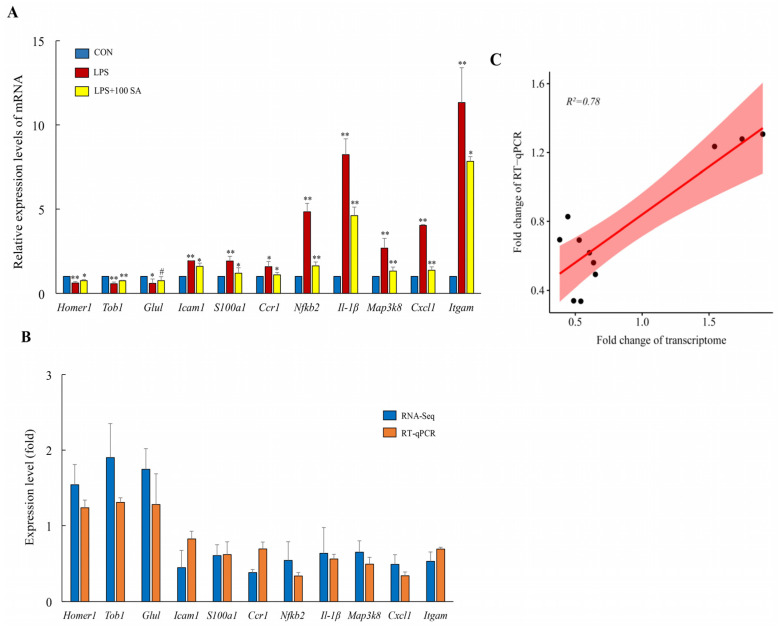
**SA reversed LPS-induced pulmonary gene expressions.** The dosage of SA in the SA group was 100 mg/kg/day, and 11 DEGs were selected for further validation through an RT-qPCR assay (**A**). Fold changes of 11 DEGs between transcriptome sequencing and the RT-qPCR assay (**B**). Linear correlation of 11 DEGs between RNA-seq and RT-qPCR (**C**). CON: control; DEGs: differentially expressed genes; LPS: lipopolysaccharide; LPS + 100 SA: LPS + 100 mg/kg/day SA; SA: sialic acid. Data are presented as the mean ± SD of three independent experiments. * *p* value was less than 0.05. ** *p* value was less than 0.01. #: *p* > 0.05.

RNA sequencing was performed to analyze the gene expression profiles of the lung tissues, which could reveal the underlying mechanism of SA against ALI. The obtained data for the genes were sequenced and filtered, and the differences among the CON group, LPS group, and LPS + 100 SA group were analyzed. The DEGs were screened, and they are described in Table 2 and Table 3. A total of 51 DEGs were identified among three groups, and the DEGs were analyzed by hierarchical clustering (Figure 4). A total of 26 upregulated genes and 25 downregulated genes were obtained by SA intake. It was significant that LPS treatment resulted in distinct differences in the gene expression profiles in lung tissues which were restored by SA. Eleven key target DEGs related to the pathogenesis of ALI were randomly selected for validation assayed by RT-qPCR. It was observed that the relative expressions of mitogen-activated protein kinase kinase kinase 8 (*Map3k8*), nuclear factor of kappa light polypeptide gene enhancer in B cells 2 (*Nfkb2*), *Il-1β*, intercellular adhesion molecule 1 (*Icam1)*, S100 calcium binding protein A11(*S100a11*), chemokine (C-C motif) receptor 1 (*Ccr1*), chemokine (C-X-C motif) ligand 1 (*Cxcl1*), and integrin subunit alpha M (*Itgam*) were downregulated 0.65, 0.54, 0.64, 0.44, 0.61, 0.38, 0.49, and 0.53 fold in the SA group using RNA sequencing and 0.49, 0.34, 0.56, 0.83, 0.62, 0.69, 0.34, and 0.69 fold in RT-qPCR, respectively. For the upregulated expression genes—homer scaffolding protein 1 (*Homer1*), glutamate-ammonia ligase (*Glul*), and transducer of ErbB-2.1 (*Tob1*)—their relative levels were increased by SA treatments at 1.54, 1.75, and 1.90 fold in RNA sequencing, and the RT-qPCR results showed that the upregulation of these genes was 1.24, 1.28 and 1.31 fold, respectively (Figure 5A,B). The above comparisons were carried out between the LPS group and SA group. The Pearson correlation coefficient of RNA sequencing and RT-qPCR was 0.78 (*p* <  0.01) for those validated genes (Figure 5C). This supported the reliability of the RNA sequencing results.

### 3.5. GO and KEGG Analyses of Identified DEGs

**Figure 6:**
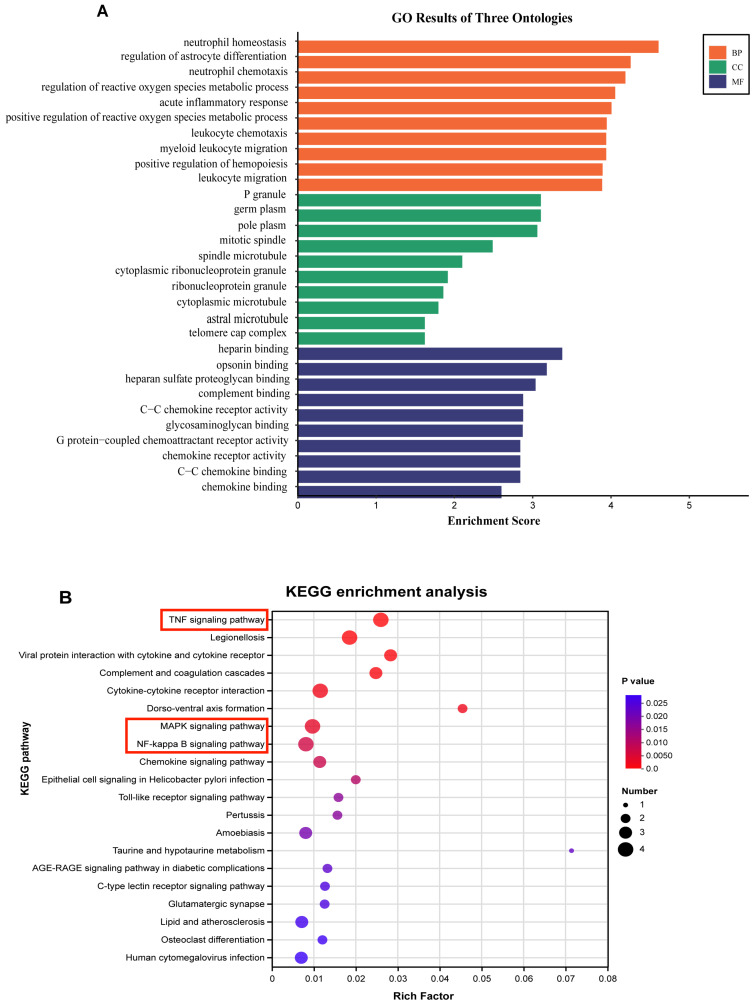
**GO enrichment and KEGG pathway analyses of identified DEGs regulated by SA.** The top GO terms enriched by 51 DEGs were uploaded into the reference list and presented in terms of BP, CC, and MF. Pathway analysis was conducted on DEGs using the KEGG database. (**A**) The top 10 terms in each category of the GO results. (**B**) The enriched pathways of the DEGs affected by SA. The top canonical pathways are marked with red boxes. BP: biological process; CC: cellular component; DEGs: differentially expressed genes; GO: gene ontology; KEGG: Kyoto Encyclopedia of Genes and Genomes; MF: molecular function; SA: sialic acid.

GO and KEGG analyses were used for further exploring the functional relevance and pathways of DEGs. The GO analysis indicated that the DEGs were associated with various functions, including the biological process (BP), cellular component (CC), and molecular function (MF). The main GO terms enriched by DEGs were relevant to neutrophil homeostasis, the regulation of reactive oxygen species metabolic process, acute inflammatory response, C-C chemokine receptor activity, chemokine receptor activity, and chemokine binding, among others (Figure 6A). These revealed terms were thought to be interrelated with inflammation and oxidation. Meanwhile, KEGG pathway analysis of the DEGs was conducted to reflect the possible pathways involved in the protection of SA in LPS-induced mice. It was found that the DEGs were enriched in the TNF signaling pathway, Legionellosis, MAPK signaling pathway, NF-κB signaling pathway, and chemokine signaling pathway, among others (Figure 6B). The presented pathways play a key impact in regulating inflammation. These results indicate that SA could modulate these signaling pathways, thereby inhibiting the expression of inflammatory factors and protecting against ALI in mice.

### 3.6. Main Network Analysis of DEGs

**Figure 7:**
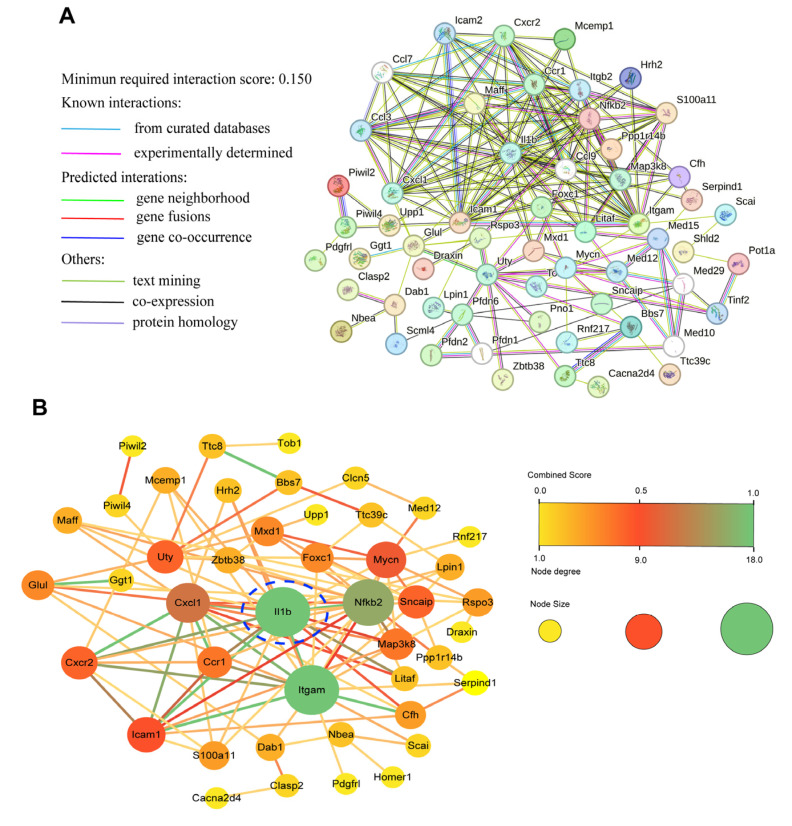
**PPI network of identified DEGs affected by SA.** The DEGs were imported to the STRING database to form an association network by automatic comparison (**A**). The PPI network was further analyzed using Cytoscape software, and a diagram of the interactive network is presented (**B**). A node represents a protein. The node size and color were determined by the degree of DEG interaction. The combined score represents the interaction between nodes. The edge size and color were determined by the combined score. The core gene is marked with the blue dashed circle.

The protein–protein interaction (PPI) network visualizes the DEGs’ mutual relationship. PPI network analysis has been used to predict and identify core targets in disease pathogenesis. Based on the above data, a PPI network was mapped on the STRING website and further analyzed using Cytoscape software. The STRING database displayed the DEG association network (Figure 7A). Then, the PPI network was uploaded into the Cytoscape software for visualization. A total of 44 DEGs with 109 edges formed the PPI network among genes affected by SA (Figure 7B), manifesting that the regulation of these genes could be responsible for the beneficial action of SA on LPS-induced ALI. The interaction results of the DEGs are showed according to combined score, and the degrees of DEG interaction are displayed by each node’s color. It is clear that IL-1β, ITGAM, NF-κB2, CXCL1, ICAM1, synuclein, alpha interacting protein (SNCAIP), C-X-C motif chemokine receptor 2 (CXCR2), and MAP3K8 were among the proteins interacted with the most. Proteins such as IL-1β, MAP3K8, NF-κB2, CXCL1, ICAM1, GLUL, and ubiquitously transcribed tetratricopeptide repeat containing, Y-linked (UTY) have been reported to be associated with inflammation and diseases [19,26,27]. These genes were also closely related to inflammation and the MAPK-NF-κB signaling pathway, which has been proven to be a vital anti-inflammatory target [28]. It also can be seen that, apparently, IL-1β occupied the core position in the PPI network. This means SA could have an anti-inflammatory ability on LPS-induced ALI through modifying the MAPK-NF-κB pathway.

### 3.7. SA Reduced the Inflammatory Factors of Lung Tissues

**Figure 8:**
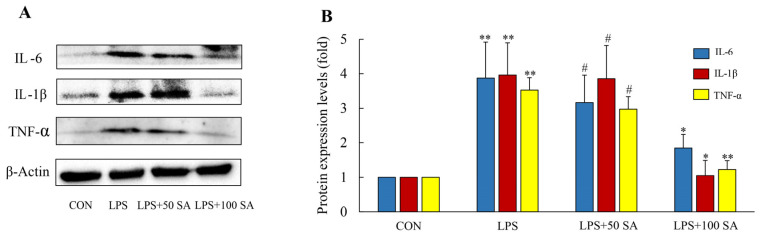
**Effects of SA supplementation on the inflammatory factors of lung tissues.** The protein levels of IL-6, IL-1β, and TNF-α of the lung tissues were measured by western blot analysis (**A**,**B**). The protein expressions of these molecules were quantified and normalized to β-actin. CON: control; LPS: lipopolysaccharide; LPS + 50 SA: LPS + 50 mg/kg/day SA; LPS + 100 SA: LPS + 100 mg/kg/day SA; SA: sialic acid. Results are presented as the mean ± SD. CON group vs. LPS group or LPS group vs. LPS + 50 SA group and LPS + 100 SA group. * *p* < 0.05. ** *p* < 0.01. # *p* > 0.05.

Inflammation generally is interrelated with dysregulated inflammatory cytokines. Cytokines like IL-6, IL-1β, and TNF-α are pro-inflammatory genes, and their levels and inflammation present a positive correlation [29]. To further verify the molecular mechanism of SA in the mitigation of ALI, related protein expressions were validated by WB analysis. Compared with the CON group, the protein levels of IL-6, IL-1β, and TNF-α were distinctly raised 3.87, 3.95, and 3.53 fold, respectively, compared with the LPS-treated group (*p* < 0.01). It is notable that SA supplementation especially blocked the generations of these inflammatory factors in a dose-dependent relation. The expressions of IL-6, IL-1β, and TNF-α were strikingly reduced by 52.20%, 73.42%, and 65.16% after 100 mg/kg SA supplementation (Figure 8A,B). As stated above, the protective effect of SA on LPS-induced mice may have contributed to its anti-inflammatory effect.

### 3.8. SA Decreased the Activations of JNK/p38/NF-κB of Lung Tissues

**Figure 9:**
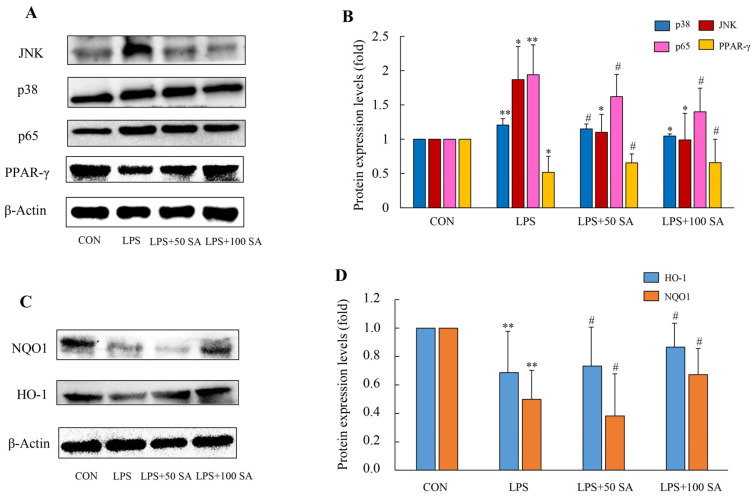
Effects of SA supplementation on the JNK/p38/PPAR-γ-NF-κB pathway and the expressions of the oxidation-related genes of the lung tissues. The protein expressions of JNK, p38, NF-κB, and PPAR-γ were tested by western blot analysis (**A**,**B**). Effects of SA supplementation on the protein levels of oxidation-related genes (HO-1 and NQO1) (**C**,**D**). CON: control; LPS: lipopolysaccharide; LPS + 50 SA: LPS + 50 mg/kg/day SA; LPS + 100 SA: LPS + 100 mg/kg/day SA; SA: sialic acid. The data are expressed as mean ± SD. CON group vs. LPS group or LPS group vs. LPS + 50 SA group and LPS + 100 SA group. * *p* < 0.05. ** *p* < 0.01. # *p* > 0.05.

The MAPK is composed of serine/threonine protein kinases formed by extracellular signal-regulated kinase (ERK) 1/2, c-Jun N-terminal kinase (JNK), and p38. NF-κB is the major nuclear transcriptional factor in the inflammatory response. Activation of the MAPK and NF-κB pathways promotes the production of inflammatory markers and aggravates inflammation [30]. As depicted in Figure 9A,B, the relative expressions of JNK and p38 were promoted markedly in the LPS-induced mice, whereas the elevated expressions of JNK and p38 were gradually restrained by SA. Also, the expression of p65 revealed a similar trend in the mice. Compared with the LPS group, treatment with 50 mg/kg and 100 mg/kg SA caused a decrease in the relative levels of p65 by about 16.49% and 27.84%, respectively. Consistent with the results of inflammatory molecule expressions, the repressions of JNK, p38, and NF-κB activation were accompanied by a reduction in pro-inflammatory molecule secretion. These results also conformed to the transcriptomic data analysis.

### 3.9. SA Promoted Activation of the PPAR-γ Signal

A series of evidence reported that peroxisome proliferator-activated receptor (PPAR) signaling mediated NF-κB inhibition [31,32]. It is capable of relieving inflammation via blocking NF-κB signaling activation, thereby diminishing the productions of downstream pro-inflammatory factors. It was found that LPS greatly restrained the PPAR-γ activity in lung tissues. Compared with the CON group, the expression of PPAR-γ was markedly decreased by 48.38% in the LPS-treated group. However, SA could reverse this change in a dose-dependent relation (Figure 9A,B). According to the above results, SA could exhibit an anti-inflammatory property via promoting PPAR-γ transactivation and inactivating the NF-κB pathway, thereby preventing inflammation.

### 3.10. SA Modulated the Oxidation-Related Gene Expressions of the Lung Tissues

Oxidative damage is an essential process in the inflammatory response [33], and the suppression of oxidative stress is key to improving ALI. Nrf2, HO-1, and NQO1 are master regulators in the antioxidant defense system [34]. As illustrated in (Figure 9C,D), LPS stimulation downregulated the protein expressions of HO-1 and NQO1 compared with the CON group. The protein levels of HO-1 and NQO1 significantly declined by 31.30% and 60.77%, respectively. However, the contents of these proteins were upregulated by SA, and the 100 mg/kg SA pretreated group led to a notable increment in their expressions. This proves that the antioxidant function of SA was able to counteract the LPS-mediated ALI.

### 3.11. Effect of SA on HUVEC Cell Viability

**Figure 10:**
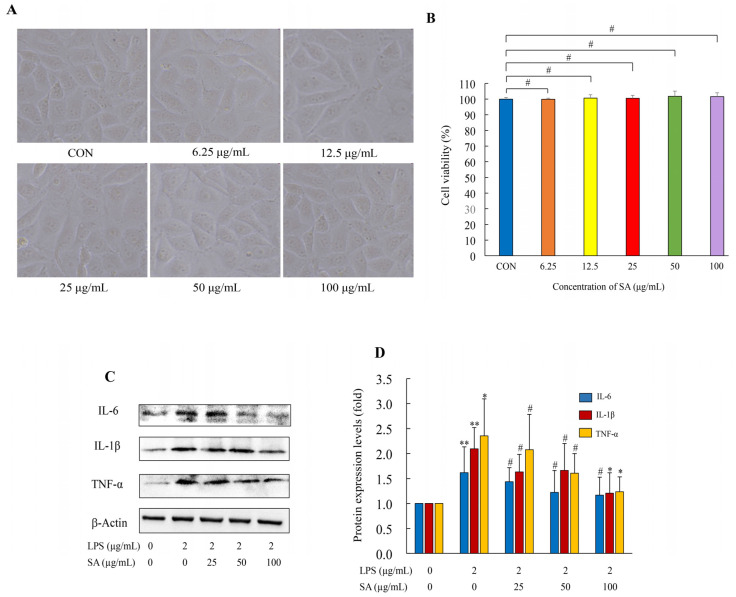
Effect of SA on the cell viabilities of HUVEC cells and the inflammatory cytokine expressions in LPS-stimulated HUVEC cells. HUVEC cells were treated with different dosages of SA (0, 6.25, 12.5, 25, 50, and 100 μg/mL), and the viability was determined by an MTS assay. The cells’ morphological features were imaged by an inverted microscope (**A**), and the viability among each group was measured by an MTS assay (**B**). HUVEC cells were pretreated with different dosages of SA (25, 50, and 100 μg/mL) for 2 h and then treated with LPS for 10–12 h. The total protein was obtained from the cells, and the protein expressions of the pro-inflammatory cytokines were determined by western blot analysis (**C**,**D**). CON: control; LPS: lipopolysaccharide; SA: sialic acid. The images shown here are the representative results from three independent experiments. Results are expressed as mean ± SD. CON group vs. LPS group or LPS group vs. 25, 50, and 100 μg/mL SA group. * *p* < 0.05. ** *p* < 0.01. # *p* > 0.05.

In the present study, the model of LPS-induced HUVEC cells was used to assess the effect of SA on ALI in vitro. In order to evaluate the optimal dose of SA on HUVEC cells with low cytotoxicity, the cells were treated with a range of concentrations (0, 6.25, 12.5, 25, 50, and 100 μg/mL). The cell viability was determined by the cell morphology and MTS assays. With the increase in SA concentration, no significant morphological changes in the HUVEC cells were observed (Figure 10A). Additionally, the MTS assay confirmed that different dosages of SA (0–100 μg/mL) had no significant difference on the cell viability among each group. The viabilities of the cells treated with different contents of SA (0–100 μg/mL) were 100.00 ± 1.03%, 99.94 ± 0.72%, 100.73 ± 2.09%, 100.65 ± 1.78%, 101.81 ± 3.38%, and 101.58 ± 2.48% (Figure 10B). These results suggest that SA has no cytotoxicity against normal HUVEC cells. Accordingly, 25, 50, and 100 μg/mL SA were selected as the experimental doses for the following experiments.

### 3.12. SA Suppressed the Inflammatory Cytokines in LPS-Induced HUVEC Cells

To explore the anti-inflammation of SA in vitro, the expressions of inflammation-related markers in HUVEC cells were estimated. WB analysis showed that the expressions of IL-6, IL-1β, and TNF-α were dramatically increased in the LPS group compared with the CON group. The relative contents of these pro-inflammatory markers in the LPS-treated group elevated 1.62, 2.09, and 2.35 fold (Figure 10C,D). SA decreased the protein levels of IL-6, IL-1β, and TNF-α in LPS-stimulated HUVEC cells in a dose–effect relation; the 100 μg/mL SA treatment effectively reduced them by 27.78%, 42.11%, and 47.23%, respectively. The decline in inflammatory molecule expression was indicative of the anti-inflammatory activity of SA. These results were in accord with the in vivo research. All of these data demonstrated that SA mitigated inflammation through repressing the pro-inflammatory cytokines’ expression.

### 3.13. SA Blocked NF-κB and AP-1 Activations

**Figure 11:**
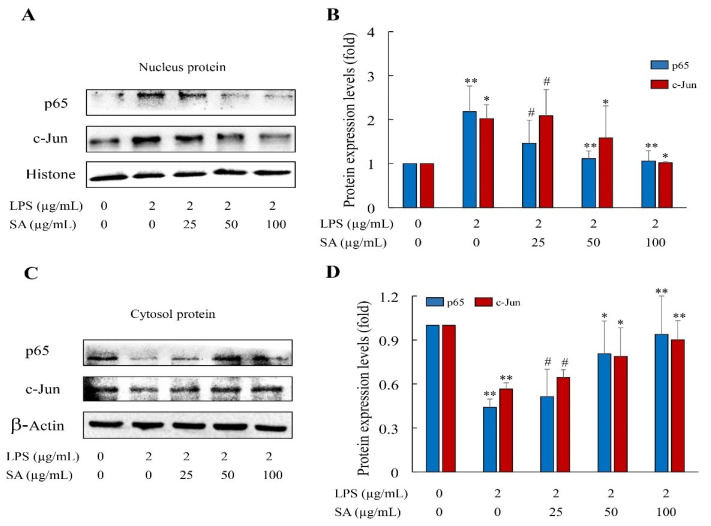
**SA inhibited the nuclear translocations of NF-κB and AP-1 in LPS-induced HUVEC cells.** The protein expressions of transcriptional regulators p65 and c-Jun in nuclear (**A**,**B**) and cytoplasmic (**C**,**D**) extracts were examined by western blot analysis. CON: control; LPS: lipopolysaccharide; SA: sialic acid. The images shown here are representative results from three independent experiments. Data are shown as mean ± SD. CON group vs. LPS group or LPS group vs. 25, 50, and 100 μg/mL SA group. * *p* < 0.05. ** *p* < 0.01. # *p* > 0.05.

NF-κB and AP-1 are two essential transcriptional regulators of pro-inflammatory molecules mediating the inflammatory process [35]. Therefore, to detect whether the activations of NF-κB and AP-1 were restrained by SA in LPS-induced HUVEC cells, the contents of transcriptional regulators p65 and c-Jun in cytoplasmic and nuclear extracts were examined by WB analysis. The results showed that stimulation with LPS increased the relative expressions of p65 and c-Jun in the cells’ nuclear proteins, and fewer p65 and c-Jun activities in the cell cytoplasmic proteins occurred, manifesting that the stimulation of LPS profoundly increased the nuclear translocations of p65 and c-Jun (Figure 11). However, SA pretreatment greatly reversed p65 and AP-1 translocations in the HUVEC cells. Consistent with the results of inflammatory factor expression, the downregulation of NF-κB and AP-1 transcriptional activations led to a decrease in pro-inflammatory factor production. Collectively, the prevention of NF-κB and AP-1 activities may be correlated with the anti-inflammatory property of SA.

### 3.14. SA Inhibited JNK and p38 Activations In Vitro

**Figure 12:**
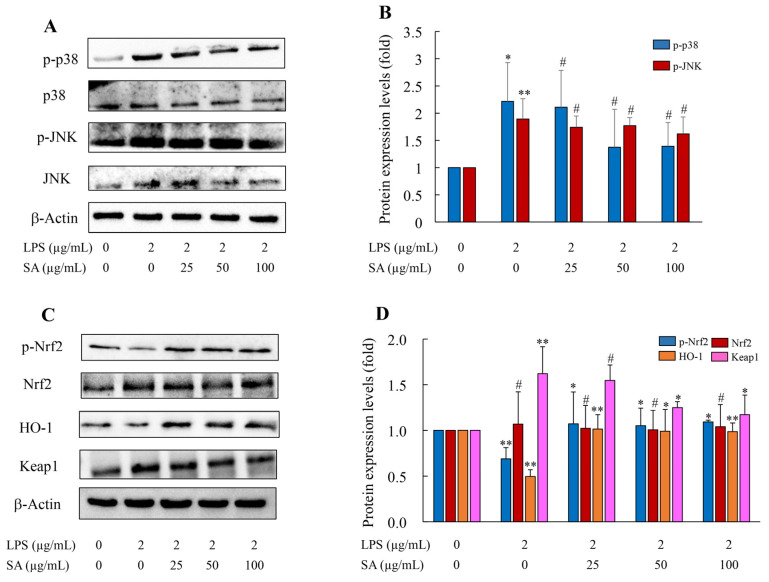
**Effects of SA supplementation on the p38/JNK and Nrf2 pathways in LPS-induced HUVEC cells.** HUVEC cells were pretreated with different dosages of SA (25, 50, and 100 μg/mL) for 2 h and then treated with LPS for 10–12 h. The total protein was obtained from the cells, and the protein expressions of p38, p-p38, JNK, and p-JNK were determined by western blot analysis (**A**,**B**). The effects of SA supplementation on the Nrf2 pathway (**C**,**D**). LPS: lipopolysaccharide; SA: sialic acid. Data are presented as the mean ± SD of three independent experiments. CON group vs. LPS group or LPS group vs. 25, 50, and 100 μg/mL SA group. * *p* < 0.05, ** *p* < 0.01. # *p* > 0.05.

The MAPK signaling cascade has a critical impact on inflammation. In the inflammatory setting, the MAPK pathway is activated and phosphorylated, resulting in activation of the NF-κB or AP-1 signal. NF-κB and AP-1 activations in turn promote inflammatory mediator generation [36]. To identify the anti-inflammatory mechanism of SA in LPS-induced HUVEC cells, activation of the MAPK pathway was further explored via WB analysis. Compared with the CON group, the protein levels of p-JNK and p-p38 could be elevated upon LPS treatment. The relative levels of p-JNK and p-p38 were increased 1.89 and 2.21 fold when stimulated by LPS, respectively, with no difference in expression for the total JNK and p38 (Figure 12A,B). As expected, SA repressed the phosphorylation of JNK and p38. From these data, SA may exhibit anti-inflammatory action through the regulation of the JNK/p38 pathway in vitro.

### 3.15. SA Reduced Oxidative Damage via the Nrf2 Pathway

The Nrf2 pathway functions as a defense mechanism, modulating the activations of antioxidative genes and ultimately exerting an anti-inflammatory function [37,38]. In response to extracellular stimulations, the activated Nrf2 signal interacts with Keap1 and further promotes HO-1 induction. In this work, LPS treatment reduced the phosphorylation level of Nrf2, which means LPS caused oxidative damage through the Nrf2 pathway. However, SA treatments (25, 50, and 100 μg/mL) upregulated the p-Nrf2 level in a dosage-dependent manner. As for Keap1, the negative regulator of Nrf2, its expression was increased apparently in the LPS group. Pretreatment of SA potently reduced Keap1 generation when compared with the LPS group. Also, HO-1 expression was downregulated when the HUVEC cells where exposed to LPS. Its protein expression was notably reduced by 50.51%. On the contrary, SA treatments upregulated their levels in a dosage-dependent manner (Figure 12C,D); that is, SA could prevent LPS-caused damage through the Nrf2-Keap1-HO-1 pathway.

## 4. Discussion

**Figure 13:**
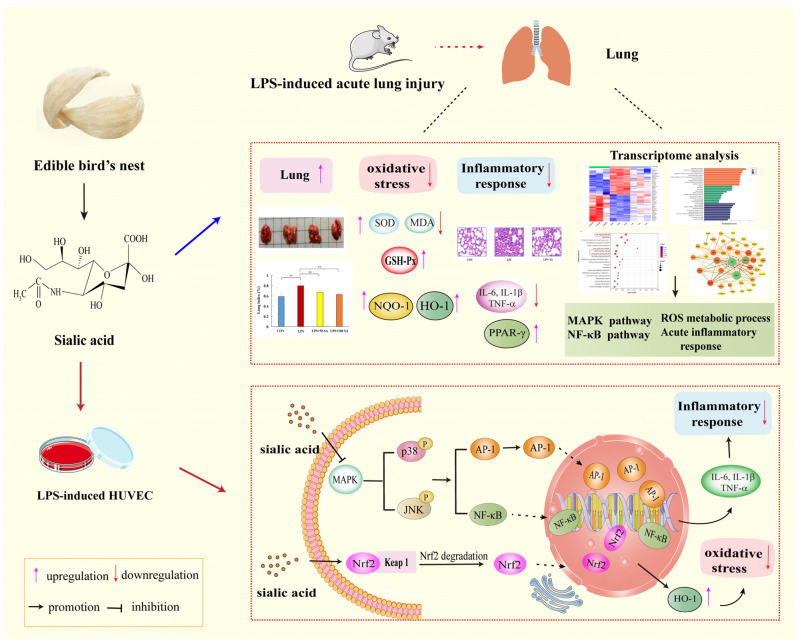
**Schematic representation of the protective effect and possible molecular mechanisms of SA on ALI.** SA exerted a protective effect against LPS-induced ALI via ameliorating inflammation and oxidative damage, which worked mainly through regulation of the JNK/p38-NF-κB/AP-1 and Nrf2 signaling pathways. ALI: acute lung injury; HUVEC: human umbilical vein endothelial cells; LPS: lipopolysaccharide; SA: sialic acid.

ALI is a serious pulmonary disease which is interrelated with ARDS. Due to its high mortality and morbidity, more attention has been paid to the prevention of ALI in recent years [3,39]. As a typical nutrient component of EBN, SA is reported to have multiple biological functions contributing to health benefits [17,40,41]. Our previous study confirmed its beneficial effect on ulcerative colitis [19]. However, there have been no investigations which linked SA actions to ALI. In this research, a systems analysis approach was performed to explore the protective function and correlated underlying mechanism of SA in ALI (Figure 13). SA supplementation could attenuate LPS-induced lung injury in mice through inhibiting inflammation and oxidative stress. In parallel, the regulation of SA treatment in LPS-treated HUVEC cells was examined, which verified its better anti-inflammatory and antioxidative abilities by modulating the JNK/p38-NF-κB/AP-1 and Nrf2 signaling pathways. This was the first time the beneficial effect of SA in the prevention of ALI was investigated.

LPS is recognized as an effective activator of ALI, and related studies have demonstrated that oxidative stress and inflammation are closely related to ALI [42]. MDA is a product of excessive reactive oxygen species (ROS) accumulation, and it reveals destruction of the epithelium. SOD and GSH-Px are enzymatic antioxidants scavenging free radicals. MDA, SOD, and GSH-Px reflect the degree of oxidative damage and serve as the central established biomarkers for ALI [43]. LPS treatment promoted ROS production and decreased antioxidant levels, thereby exacerbating oxidative stress in lung tissues. SA supplementation prominently promoted the activities of SOD and GSH-Px and restrained the elevation of the MDA level in LPS-induced mice. The activated oxidative stress is often accompanied by severe inflammatory conditions in ALI [44]. The protection of SA against pulmonary inflammation was also presented through H&E staining. For the above, SA intervention could improve lung function and relieve oxidative injury and inflammation in LPS-induced mice.

The prevention of ALI may be attributed to the regulation of target gene expression. To further elucidate the molecular mechanisms of SA in the protection of ALI, RNA-seq transcriptome analysis was subsequently executed. In this study, a total of 51 DEGs were identified, and GO analysis indicated that these genes enriched functions, including neutrophil homeostasis, regulation of reactive oxygen species’ metabolic process, acute inflammatory response, and chemokine receptor activity. These terms demonstrated that oxidative stress and inflammation were dominantly involved in ALI, similar to the above results. Oxidative stress resulting from excessive ROS accumulation promoted the occurrence of inflammation in response to LPS stimuli, and it aggravated lung damage [45]. DEGs mapped to KEGG pathways predicted that SA could modulate the TNF, MAPK, and NF-κB signaling pathways. The TNF signaling pathway plays a crucial part in mammalian immunity. It is mainly associated with intracellular homeostasis and inflammatory pathology [46]. It has been established that MAPK and NF-κB signaling cascades act as the canonical pathways in the inflammatory process [47,48]. Stimulated with extracellular stimuli, the activated MAPK augmented the activation of transcription factor NF-κB, followed by the release of inflammatory cytokines. Series of studies proved that TNF, MAPK, and NF-κB signaling could be the potential targets of various bioactive constituents in the improvement of lung injuries [49,50]. These findings collectively show that SA could attenuate oxidative stress and inflammatory reactions in ALI through modulation of the TNF, MAPK, and NF-κB pathways.

Inflammation generally involves the overexpression of pro-inflammatory cytokines, including IL-6, IL-1β, and TNF-α. They recruit the immune cells implicated in the etiology of various inflammatory conditions. PPI network analysis revealed that IL-1β was the key protein, and it was a critical member of the inflammatory factors. In this work, the pulmonary IL-1β level in the LPS group was higher than that in the CON group. Also, SA intervention could reduce inflammation by lowering the levels of IL-6, IL-1β, and TNF-α in a dose-dependent relation. Our previous study demonstrated the inhibitory effect of SA on the colitis via diminishing pro-inflammatory gene expression [19]. In this regard, SA exerted a protective function through the inhibition of inflammatory gene secretion in ALI. In the meantime, the production of JNK, p38, and p65-NF-κB greatly increased in the presence of LPS. Supplementation of SA could negatively regulate the activities of these cytokines, which means SA played a protective role by interfering with JNK/p38-NF-κB activations in ALI. These data were able to validate the transcriptome analysis. Along with MAPK and NF-κB, PPAR-γ is another significant pathway for mediating inflammatory reactions. It is capable of blocking NF-κB activation and lowers the production of pro-inflammatory mediators [51]. Our results determined that SA supplementation significantly increased the activation of PPAR-γ, and the LPS + 100 SA group presented the higher PPAR-γ expression in the lung tissues. Lin et al. [52] reported that magnolol apparently improved ALI and downregulated pro-inflammatory cytokine contents by inactivating the MAPK and NF-κB signals and upregulating PPAR-γ, which is consistent with our research. In light of these data, SA exhibited anti-inflammatory action largely depending upon modulation of the JNK, p38, and PPAR-γ-NF-κB signaling pathways.

Furthermore, oxidative stress is tightly correlated with ALI. Nrf2 is a crucial regulatory transcription factor, and the Nrf2 pathway is considered the pivotal antioxidant mechanism and confers a protective role against oxidative injury [53,54]. In general, Nrf2 is inactive when combined with Keap1 in the cytoplasm. When stimulated by LPS, oxidative stress, and other stimuli, Nrf2 can be activated. After activation, it dissociates from Keap1 and enters the nucleus to trigger the release of related downstream cytokines like HO-1 and NQO1 [55,56]. In this study, the expressions of HO-1 and NQO1 decreased greatly in the LPS-stimulated lung tissues but were enhanced significantly when pre-treated with SA. This means that SA had an excellent antioxidant property for defending against ALI.

Vascular endothelial cells attach to the lining of blood vessels and spread all over the circulatory system, which mediate a range of immune responses. Growing evidence demonstrated that SA could regulate cell–cell interaction among endothelial cells in the prevention of diseases [57,58]. As a result, to further explore the protective effect and mechanism of SA against ALI, LPS was used to induce HUVEC in vitro. It was observed that SA successfully diminished the expression of pro-inflammatory genes such as IL-6, IL-1β, and TNF-α. Hu et al. [57] also reported that SA could reduce LPS-driven inflammatory cytokine productions (IL-6 and TNF-α) in HUVEC. These data are indicative of the anti-inflammatory activity of SA due to the decline in inflammatory gene expression.

NF-κB and AP-1 are well known to be the key regulatory transcription factors aggravating the secretion of pro-inflammatory mediators. The anti-inflammatory capacity of many compounds is related to the inactivation of NF-κB and AP-1 [59]. NF-κB and AP-1 are closely integrated with inhibitory proteins. The stimuli activate NF-κB and AP-1, and they enter the nucleus. Then, free NF-κB and AP-1 bind to the promoters of inflammatory cytokines in the nucleus, followed by the occurrence of inflammation [60]. In the current study, the translocation levels of NF-κB and AP-1 were gradually decreased by SA in LPS-stimulated HUVEC. Our previous study also suggested that SA strongly inhibited the luciferase activities of NF-κB and AP-1 in LPS-induced RAW264.7 cells [19]. Hence, NF-κB and AP-1 inactivation could account for the anti-inflammatory capacity of SA.

MAPK, a group of protein kinases including ERK1/2, JNK, and p38, is the pivotal cellular signal which mediates numerous physiological processes. Upon activation, MAPK could be phosphorylated, and p-ERK1/2, p-JNK, and p-p38 can target NF-κB or AP-1 transcriptional activity, thereby regulating inflammatory molecule expressions. Publications highlighted the beneficial role of the MAPK signaling cascade in the attenuation of ALI [42,49]. In this perspective, our data implied that the inhibitory activity of SA on p-JNK and p-p38 underlie its anti-ALI effect. Moreover, 23-O-acetylshengmanol-3-o-α-L-arabinoside plays an ALI-protective role, as it restrained the nuclear translocation of p65 and AP-1, as well as the phosphorylation of IκBα, ERK, and p38 [61]. Mechanistically, it validated the role of the MAPK-NF-κB and AP-1 pathways in the treatment of ALI. In short, the anti-ALI mechanism of SA might be attributed to repression of the p-JNK- and p-p38-mediated activities of NF-κB and AP-1 signals, resulting in mitigation of the inflammatory response.

It was recognized that oxidative stress was a central contributing factor in ALI, and it could be a promising therapeutic strategy for ALI. To verify the underlying mechanism of SA in the amelioration of ALI, the antioxidant activity of SA was investigated in LPS-induced HUVEC. Our results revealed that SA eliminated oxidative stress induced by LPS. The Nrf2 pathway is the notable anti-oxidant target involved in cell protection. Under oxidative stress and inflammatory conditions, massive induction of HO-1 and a decline in Keap1 can protect the cell from ROS accretion. Glycocalyx SA was reported to possess antioxidant activity via modulation of the Nrf2 signal, upregulating the expressions of HO-1 and NQO1 in the HUVEC [62]. Our findings were identical with these results in that SA modified Nrf2 signal-related genes, including HO-1 and Keap1, in the HUVEC. Similarly, sitagliptin defended against oxidative damage and excessive autophagy in ALI through regulating the p62-Keap1-Nrf2 signaling pathway [63]. In summary, SA might have a potential anti-oxidant effect by modifying the activation of the Nrf2-Keap1-HO-1 pathway in LPS-stimulated HUVEC.

## 5. Conclusions

ALI is a kind of lung disease which seriously affects human health. Natural products have attracted more and more attention because of their low toxicity or non-toxicity. Using natural products to prevent and treat ALI is an important development direction in the future. This study suggests that SA could relieve the symptoms of ALI through its anti-inflammation and anti-oxidation properties and confirms the molecular mechanism of SA by regulating the JNK/p38-NF-κB/AP-1 and NRF2 signaling pathways. These findings provide new clues for the prevention and treatment of ALI. However, with this study on the short-term effects of SA on ALI, future work still needs to study the long-term effects of SA on ALI. In particular, our current work only had initial animal experiments, and much more clinical practice is needed to become the prevention and treatment of ALI in clinics. Although we studied the molecular mechanism of action, can SA affect the gut microbiota and metabolites? How do these metabolites regulate signaling pathways and the expression of related genes? This is not clear yet, and further research is needed. Some glycoproteins can be absorbed directly, but SA may also be degraded in the digestive tract. Do these degradation products play a role in anti-inflammatory function? Whether these degradation products can affect changes in the gut microbiota and metabolites needs to be further explored.

## Figures and Tables

**Table 1:** The primer sequences used for RT-qPCR analysis.

Gene Name	Primer Sequence (5′-3′)	Primer Length (bp)
*β-actin*	F: TCACTATTGGCAACGAGCGGTTC	23
*β-actin*	R: AGCACTGTGTTGGCATAGAGGTCT	24
*IL-1β*	F: GAG CAC CTT CTT TTC CTT CAT CTT	24
*IL-1β*	R: TCA CAC ACC AGC AGG TTA TCA TC	23
*Homer1*	F: GGAGAAGTCGCAGGAGAAGATG	22
*Homer1*	R: GCTGATTGCTGAACTATGTGGAA	23
*Glul*	F: GCAGAGACCAACTTGAGGCACATC	24
*Glul*	R: GCTCCCACACCGCAGTAATACG	22
*Tob1*	F: CCACCAAGTTCGGCTCCACCAA	22
*Tob1*	R: TCTGCTTCAGGAGGTCGTTCACATT	25
*S100a11*	F: TGGTGTCCTTGACCGCATGATGAA	24
*S100a11*	R: GGAGGTGATGACTTGGTGGTTGGAT	25
*Ccr1*	F: GAAGGTCAAAGCCGTGCGTCTG	22
*Ccr1*	R: GGTCCAGTTGCTTACTCTGCTCACA	25
*Itgam*	F: GGAGCATCAATAGCCAGCCTCAGT	24
*Itgam*	R: ACAGCCAGGTCCATCAAGCCATC	23
*Cxcl1*	F: ACCGAAGTCATAGCCACACTCAAGA	25
*Cxcl1*	R: AGAAGCCAGCGTTCACCAGACA	22
*Icam1*	F: GGAGACGCAGAGGACCTTAACAGT	24
*Icam1*	R: CGCCGCTCAGAAGAACCACCTT	22
*Nfkb2*	F: GCACAGGACGAGAACGGAGACA	22
*Nfkb2*	R: GCAGGTGGTTGGTGAGGTTGATG	23
*Map3k8*	F: CACAGGCAGCACCGAAGAGTCT	22
*Map3k8*	R: AAGCCATCCATCAGCCGTATTCCA	24

**Table 2:** Genes upregulated by SA from RNA-Seq.

Gene ID	Gene Name	Fold Change LPS/CON	*p* Value LPS/CON	Fold Change LPS + 100 SA/LPS	*p* Value LPS + 100 SA/LPS
ENSMUSG00000068457	*Uty*	0.39	0.0199	1.51	0.0107
ENSMUSG00000037325	*Bbs7*	0.41	0.0091	1.52	0.0181
ENSMUSG00000063760	*Rnf217*	0.45	0.0048	1.52	0.0003
ENSMUSG00000007617	*Homer1*	0.57	0.0056	1.54	0.0262
ENSMUSG00000029676	*Pot1a*	0.42	0.0348	1.58	0.0357
ENSMUSG00000033392	*Clasp2*	0.54	0.0437	1.63	0.0380
ENSMUSG00000026365	*Cfh*	0.40	0.0247	1.64	0.0464
ENSMUSG00000026473	*Glul*	0.55	0.0062	1.75	0.0115
ENSMUSG00000041471	*Shld2*	0.41	0.0002	1.80	0.0143
ENSMUSG00000021013	*Ttc8*	0.17	0.0136	1.81	0.0432
ENSMUSG00000037573	*Tob1*	0.16	0.0453	1.90	0.0485
ENSMUSG00000020593	*Lpin1*	0.20	0.0407	1.94	0.0106
ENSMUSG00000040433	*Zbtb38*	0.26	0.0329	2.08	0.0342
ENSMUSG00000050295	*Foxc1*	0.22	0.0156	2.22	0.0247
ENSMUSG00000027799	*Nbea*	0.22	0.0411	2.29	0.0147
ENSMUSG00000024534	*Sncaip*	0.19	0.0012	2.29	0.0398
ENSMUSG00000024424	*Ttc39c*	0.37	0.0373	2.30	0.0233
ENSMUSG00000031595	*Pdgfrl*	0.07	0.0301	2.35	0.0172
ENSMUSG00000033900	*Map9*	0.17	0.0471	2.36	0.0313
ENSMUSG00000004317	*Clcn5*	0.33	0.0428	2.70	0.0371
ENSMUSG00000036912	*Piwil4*	0.17	0.0352	2.90	0.0124
ENSMUSG00000035236	*Scai*	0.31	0.0314	3.16	0.0026
ENSMUSG00000019880	*Rspo3*	0.20	0.0014	3.18	0.0030
ENSMUSG00000029005	*Draxin*	0.15	0.0353	3.29	0.0411
ENSMUSG00000044770	*Scml4*	0.13	0.0489	3.61	0.0292
ENSMUSG00000028519	*Dab1*	0.08	0.0260	4.34	0.0303

**Table 3:** Genes downregulated by SA from RNA-Seq.

Gene ID	Gene Name	Fold Change LPS/CON	*p* Value LPS/CON	Fold Change LPS + 100 SA/LPS	*p* Value LPS + 100 SA/LPS
ENSMUSG00000034987	*Hrh2*	19.92	0.0160	0.27	0.0402
ENSMUSG00000037169	*Mycn*	3.88	0.0039	0.34	0.0165
ENSMUSG00000022766	*Serpind1*	1.88	0.0271	0.38	0.0315
ENSMUSG00000025804	*Ccr1*	7.47	0.0046	0.38	0.0148
ENSMUSG00000033644	*Piwil2*	2.95	0.0456	0.42	0.0481
ENSMUSG00000026180	*Cxcr2*	20.07	0.0001	0.44	0.0500
ENSMUSG00000037405	*Icam1*	1.85	0.0055	0.44	0.0245
ENSMUSG00000029380	*Cxcl1*	11.63	0.0000	0.49	0.0032
ENSMUSG00000020407	*Upp1*	6.37	0.0000	0.52	0.0078
ENSMUSG00000030786	*Itgam*	14.29	0.0001	0.53	0.0077
ENSMUSG00000025225	*Nfkb2*	3.02	0.0026	0.54	0.0475
ENSMUSG00000024309	*Pfdn6*	2.57	0.0099	0.57	0.0450
ENSMUSG00000001156	*Mxd1*	4.06	0.0035	0.57	0.0261
ENSMUSG00000041460	*Cacna2d4*	1.85	0.0011	0.58	0.0092
ENSMUSG00000027907	*S100a11*	1.96	0.0075	0.61	0.0177
ENSMUSG00000013974	*Mcemp1*	4.07	0.0042	0.61	0.0468
ENSMUSG00000020116	*Pno1*	1.50	0.0043	0.62	0.0183
ENSMUSG00000042622	*Maff*	2.50	0.0033	0.62	0.0173
ENSMUSG00000022500	*Litaf*	2.03	0.0094	0.63	0.0041
ENSMUSG00000079487	*Med12*	2.23	0.0126	0.63	0.0341
ENSMUSG00000006345	*Ggt1*	12.88	0.0014	0.63	0.0492
ENSMUSG00000024235	*Map3k8*	2.30	0.0007	0.65	0.0262
ENSMUSG00000006412	*Pfdn2*	1.82	0.0440	0.65	0.0446
ENSMUSG00000056612	*Ppp1r14b*	3.07	0.0010	0.66	0.0243
ENSMUSG00000027398	*Il-1β*	16.63	0.0006	0.64	0.0498

## Data Availability

The original contributions presented in this study are included in the article, and further inquiries can be directed to the corresponding authors.

## Abbreviations

ALI: acute lung injury; ARDS: acute respiratory dysfunction syndrome; BCA: bicinchoninic acid; BP: biological process; CC: cellular component; Ccr1: chemokine (C-C motif) receptor 1; Cxcl1: chemokine (C-X-C motif) ligand 1; CXCR2: C-X-C motif chemokine receptor 2; DEGs: differentially expressed genes; EBN: edible bird’s nest; ERK: extracellular signal-regulated kinase; FBS: fetal bovine serum; Glul: glutamate-ammonia ligase; GO: gene ontology; GSH-Px: glutathione peroxidase; H&E: hematoxylin and eosin; HO-1: heme oxygenase-1; Homer1: homer scaffolding protein 1; HRP: horseradish peroxidase; HUVEC: human umbilical vein endothelial cells; Icam1: intercellular adhesion molecule 1; IL-1β: interleukin-1β; IL-6: interleukin-6; JNK: c-Jun N-terminal kinase; Keap1: kelch-like ECH-associated protein 1; KEGG: Kyoto Encyclopedia of Genes and Genomes; LPS: lipopolysaccharide; MAPK: mitogen-activated protein kinase; MDA: malondialdehyde; MF: molecular function; Neu5Ac: N-acetylneuraminic acid; NF-κB: nuclear factor kappa-light-chain-enhancer of activated B cells; NQO1: NADH quinone oxidoreductase 1; Nrf2: nuclear factor erythroid-2-related factor 2; PPAR: peroxisome proliferator-activated receptor; PPI: protein–protein interaction; RIPA: radioimmunoprecipitation assay; ROS: reactive oxygen species; RT-qPCR: real-time quantitative polymerase chain reaction; S100a11: S100 calcium binding protein A11; SA: sialic acid; SDS: sodium dodecyl sulfate; SNCAIP: synuclein, alpha interacting protein; SOD: superoxide dismutase; TNF-α: tumor necrosis factor-α; Tob1: transducer of ErbB-2.1; WB: western blot.
